# AI-guided mobile health integrated with physical education improves physical activity and psychophysiological outcomes in university students: a randomized controlled trial protocol

**DOI:** 10.3389/fdgth.2026.1862894

**Published:** 2026-10-21

**Authors:** Hongli Yu, Gang Xu, Xia Li, Guangwen Song, Mingmao Li, Guoping Qian, Zbigniew Ossowski, Anna Szumilewicz

**Affiliations:** 1 College of Physical Education, Sichuan University of Science and Engineering, Zigong, China; 2 Faculty of Physical Culture, Gdansk University of Physical Education and Sport, Gdansk, Poland

**Keywords:** health-related physical fitness, mobile health, physical activity, physical education, randomized controlled trial, university students

## Abstract

**Background:**

Physical inactivity among university students represents a growing global health concern, contributing to the onset of non-communicable diseases and poor physical fitness. Despite advances in mobile health (mHealth) technologies, few interventions have been effectively integrated into formal physical education curricula. The Dreamer® application was developed to address this gap by combining artificial intelligence (AI)-guided personalization with teacher-supported engagement.

**Methods:**

This randomized controlled trial aims to assess the effectiveness of Dreamer® in improving physical activity levels, health-related fitness, and psychological well-being among university students. A total of 128 participants will be randomly allocated to either an intervention group using Dreamer® integrated into PE instruction or a control group receiving standard PE. Assessments occur at baseline, 16 weeks, 32 weeks, and 3-month follow-up. Outcomes include physical activity, physical fitness, psychological health, and app usability.

Discussion: This study will be among the first to evaluate an AI-supported, teacher-supervised mHealth intervention embedded in a university PE curriculum. The findings will inform the design of scalable digital health strategies that bridge technology and pedagogy in higher education.

**Clinical Trial Registration:**

https://www.chictr.org.cn/showproj.html?proj=284314, identifier ChiCTR2600133726.

## 1 Introduction

Physical inactivity continues to pose a significant global public health challenge, contributing markedly to the prevalence of non-communicable diseases, including cardiovascular disorders, type 2 diabetes mellitus, and various forms of cancer (1–3). According to recent estimates by the World Health Organization (WHO), approximately 1.8 billion adults are at heightened risk of developing health conditions associated with inadequate physical activity (PA), with young adults representing one of the most vulnerable demographic groups (4). The transition from high school to university often marks a critical period of behavioral change during which PA levels sharply decline (5). This decline is attributed to increased academic demands, new social environments, and a shift toward more sedentary lifestyles (6). Despite extensive evidence demonstrating the benefits of regular PA—including improvements in cognitive function, mental health, and cardiovascular fitness—many university students fail to meet international PA recommendations (7).

The WHO's Global Action Plan on Physical Activity 2018–2030 advocates for a 15% worldwide reduction in physical inactivity by fostering supportive environments that encourage active lifestyles across various sectors, including education (8, 9). Within this framework, universities play a pivotal role in fostering lifelong PA habits. However, traditional physical education (PE) programs often struggle to maintain student motivation beyond structured class time and frequently lack mechanisms for personalized feedback and continuous engagement (10).

The rapid proliferation of digital and mobile technologies offers a transformative opportunity to reimagine PE and health promotion strategies. Over the past decade, mobile health (mHealth) interventions have emerged as promising tools for encouraging behavior change through accessible, data-driven feedback (11, 12). These applications typically integrate self-monitoring, goal setting, and motivational prompts, leveraging ubiquitous smartphone use among young adults to deliver interventions at scale. Nevertheless, the direction of current mHealth research has been somewhat fragmented. While systematic reviews consistently show short-term gains in step count and moderate-to-vigorous activity levels, the long-term effectiveness and behavioral sustainability of these interventions remain uncertain due to high attrition and declining user engagement (13, 14).

Furthermore, most prior studies have targeted general or clinical populations, with limited focus on university students within formal educational settings (12, 15, 16). Few interventions have been embedded directly into PE curricula, where the potential for integrating digital tools with structured instruction remains underexplored (17, 18). This represents a significant gap, as educational settings provide a controlled yet scalable environment for implementing and evaluating technology-assisted PA programs (19, 20).

Another major limitation of prior research concerns the lack of human interaction and adaptive feedback in purely app-based interventions (21). Many fitness applications rely solely on self-directed engagement, which tends to decline over time. Recent studies suggest that integrating human facilitation—such as teacher or coach involvement—into mHealth systems can enhance accountability and adherence (11). Similarly, advances in artificial intelligence (AI) have enabled the development of adaptive algorithms that personalize exercise recommendations based on individual data patterns, potentially addressing the “one-size-fits-all” limitation of earlier digital health models. However, few randomized controlled trials (RCTs) have empirically examined the combined effect of AI-driven personalization and instructor guidance within a higher education context.

In response to these research gaps, the present study introduces Dreamer®, an intelligent smartphone-based fitness application designed to integrate AI-guided exercise recommendations with teacher-supported monitoring within university PE programs. The app utilizes user health data to generate individualized exercise prescriptions, provide real-time feedback, and foster long-term engagement through gamified and social features. Teachers, in turn, supervise progress, offer personalized feedback, and provide motivational support—bridging the divide between digital autonomy and human accountability.

This protocol outlines a RCT designed to assess the effectiveness of Dreamer® in enhancing PA, health-related fitness, and psychological well-being among university students. By combining adaptive mobile technology with structured pedagogical supervision, the trial aims to address the limitations of prior mHealth interventions and contribute to the evolving paradigm of technology-enhanced PE. The findings are expected to inform evidence-based strategies for integrating mHealth tools into higher education, supporting the WHO's goal of promoting active, health-literate societies through digital innovation.

## 2 Methods

### 2.1 Design

This study adopts a two-arm, parallel-group RCT design to assess the effectiveness of the Dreamer® mHealth application in enhancing PA and health-related fitness outcomes among university students. Assessments will be conducted at baseline, post-intervention (16 weeks), 32 weeks post-randomization, and at a 3-month follow-up (Figure 1). This trial is implemented in alignment with the Standard Protocol Items: Recommendations for Interventional Trials (SPIRIT) 2013 Statement (22) and reported following the CONSORT-EHEALTH checklist for digital health interventions (23).

**Figure 1:**
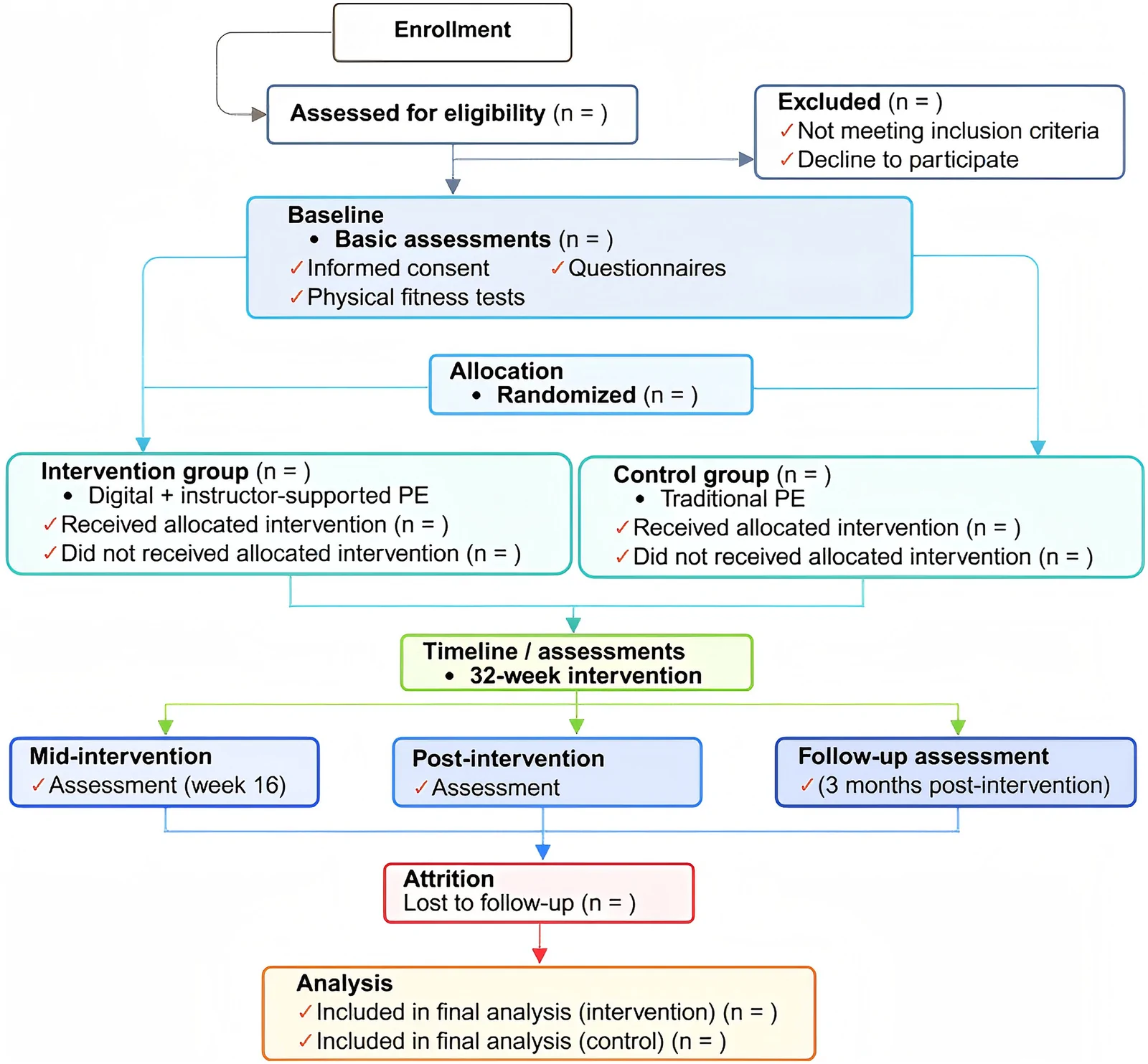
CONSORT flow diagram depicting participant recruitment, baseline evaluation, random allocation, follow-up procedures, and data analysis in the randomized controlled trial of the smartphone-based fitness application.

### 2.2 Participants and recruitment

Participants are undergraduate students enrolled in mandatory PE courses. Recruitment occurs at the beginning of the academic semester through digital invitations distributed by university administration and instructor announcements during PE orientation sessions. Eligible participants must: (1) Be full-time university students aged 18–25 years; (2) Own a smartphone compatible with Android or iOS systems; (3) Be capable of performing moderate-intensity PA; (4) be willing and able to provide written informed consent. Students with chronic illnesses, musculoskeletal limitations, or any medical condition precluding regular exercise participation are excluded. Recruitment continues until the required sample size is achieved.

The university administration will distribute initial recruitment emails detailing the study to prospective participants. Interested students can register through the study's official website, respond directly to the email inquiry, or contact the research team via phone or email. To enhance recruitment efforts, two reminder emails will be dispatched at intervals of two and four weeks following the initial communication. Students who choose not to respond will remain anonymous to the research team throughout the process.

Following the provision of informed consent, participants will undergo baseline assessments to collect initial data. These measures include socio-demographic data, a web-based questionnaire assessing self-reported physical activity, metal health outcomes, and attitudes towards fitness technology. Participants will be instructed to wear a pedometer or fitness tracker for seven consecutive days to obtain objective measurements of PA levels. During this period, individuals in the intervention group will be instructed to download and install the Dreamer® mobile application.

### 2.3 Randomization and blinding

Participants are randomly allocated in a 1:1 ratio to either the intervention group, which receives digitally enhanced and instructor-supported physical education, or the control group, which receives standard physical education instruction. Randomization sequences are computer-generated by an independent statistician using block randomization stratified by gender and baseline PA level. Allocation concealment is ensured through sequentially numbered, opaque, sealed envelopes. Given the behavioral nature of the intervention, participant blinding is not feasible; however, data collectors and analysts remain blinded to group assignment to minimize assessment bias.

### 2.4 Intervention

Experiment group (Digital + Instructor-Supported PE): Participants assigned to the intervention group will utilize the Dreamer® app continuously over two consecutive semesters, corresponding to an approximate duration of 32 weeks. The app integrates mHealth and artificial intelligence (AI)-driven analytics to personalize exercise recommendations based on user data (e.g., body composition, step count, and activity frequency). Its core components include:

1. Online course management
  Participants will use the app to register for PE classes, sign in for attendance, and track their participation. The app facilitates streamlined course management for both students and instructors, ensuring accurate monitoring of engagement (Figure 2).
2. Data-driven personalized exercise recommendations and health monitoring
  The application collects and manages participants' exercise-related and physical fitness information. In particular, participants' physical fitness assessment results are incorporated into the recommendation function to provide differentiated exercise guidance according to individual fitness profiles. Based on these data, the system pushes exercise content and recommendations appropriate to the participant's current physical fitness status and identified exercise needs. Therefore, participants with different physical fitness profiles may receive different exercise recommendations rather than a uniform exercise program.
  The detailed proprietary computational architecture and source code underlying the recommendation function are inaccessible to the research team. Accordingly, the present protocol describes the observable data inputs and recommendation functions implemented in the intervention rather than attributing the system to a specific machine-learning architecture (Figure 2).
3. Campus running program
  The app includes multiple running modes, such as timed runs and distance challenges, which participants will complete throughout the semester. The app automatically logs running sessions, recording metrics such as date, distance (1–3 km per session), duration, and frequency. Progress towards the semester goal of 90 km will be tracked in real time, with in-app notifications and reminders to encourage participants to meet weekly exercise targets (Figure 2). During running sessions, the application monitors participants' running speed and provides real-time prompts when the pace is above or below the predefined target range. These prompts allow participants to adjust their running pace during exercise and provide immediate exercise-intensity feedback. Performance feedback, including pace and distance analysis, will be provided after each session, with weekly summaries offering detailed insights into progress. Motivational elements, such as achievement badges for completing milestones, will be incorporated to encourage continued engagement. The app also enables social sharing, fostering peer motivation and friendly competition.
4. Teacher involvement and monitoring
  Instructors will have access to real-time data from the app, enabling them to monitor participants' progress closely. Weekly feedback sessions will be conducted where instructors review app data with participants, providing guidance, encouragement, and personalized adjustments to help students meet their targets. If a participant falls behind in their running goals, teachers will offer additional support through both automated and personal reminders. Thus, app-generated feedback and recommendations are supplemented by instructor review rather than functioning as a fully autonomous exercise-prescription system.

**Figure 2:**
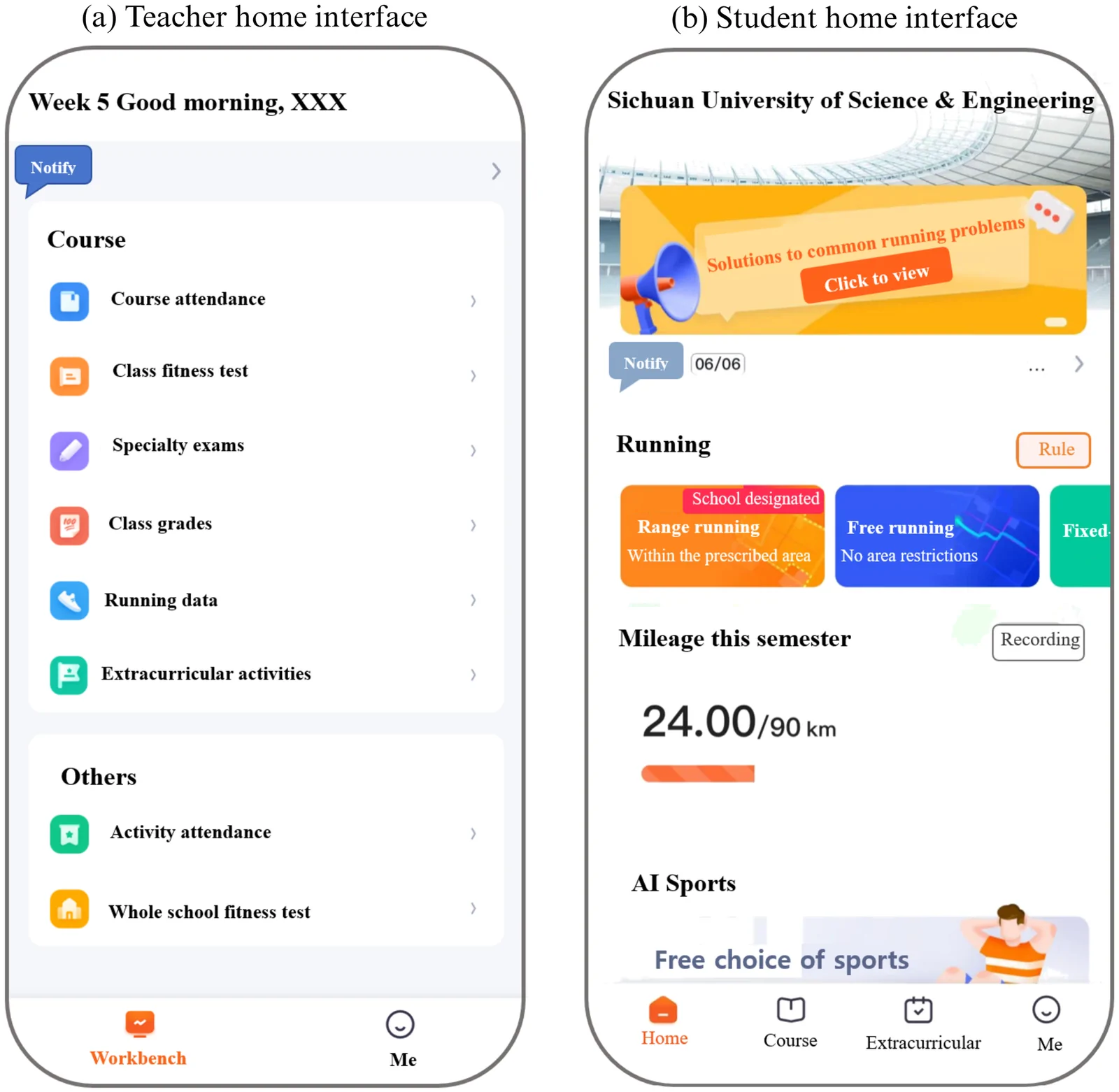
Representative teacher-facing interfaces **(a)** and student-facing **(b)** of the dreamer® mobile application.

Control group (Traditional PE): Participants assigned to the control group will engage in standard PE instruction, without incorporating the use of the Dreamer® app. They will follow a similar semester-long exercise regimen as the experimental group, aiming to complete 90 km of running across two semesters, with individual sessions limited to 1–3 km. Unlike the experimental group, control participants will not receive personalized exercise prescriptions, automated feedback, or achievement badges. They will manually record their running sessions in a physical or digital logbook, noting the date, distance, and duration for each run. Control participants will rely entirely on self-motivation, without the external support or reminders provided by the app.

### 2.5 Sample size and power considerations

The sample-size calculation was based on the prespecified primary confirmatory endpoint of PA, assessed using the International Physical Activity Questionnaire (IPAQ) at Week 32. A previous randomized trial involving college students and a mobile-supported behavioral intervention reported a between-group difference in IPAQ-assessed PA corresponding to a standardized effect of approximately Cohen's *d* = 0.56. To avoid relying on the exact effect estimate from a single previous study, a slightly more conservative standardized effect size of Cohen's *d* = 0.55 was used for the present sample-size calculation. An *a priori* power analysis was conducted using G*Power for a two-sided comparison between two independent groups. With Cohen's d = 0.55, *α* = 0.05, statistical power of 80% (1−*β* = 0.80; *β* = 0.20), and a 1:1 allocation ratio, a minimum of approximately 53 evaluable participants per group (106 participants in total) is required.

To account for an anticipated attrition rate of approximately 15%, the required recruitment sample was adjusted using N/(1−0.15), resulting in approximately 63 participants per group. The recruitment target was therefore set at 128 participants (64 per group), providing a small additional margin beyond the minimum attrition-adjusted requirement. The primary analysis will use a baseline-adjusted comparison of Week 32 PA. The sample-size calculation was intentionally based on the more conservative unadjusted between-group comparison and therefore does not rely on an assumed baseline-follow-up correlation.

### 2.6 Outcomes and measures

#### 2.6.1 Health-related physical fitness

Health-related physical fitness will be evaluated through a combination of data recorded by the app and standardized fitness testing protocols, consistent with the guidelines set forth in the ACSM's Health-Related Physical Fitness Assessment Manual (24). The assessment includes several key metrics designed to evaluate different aspects of physical fitness:

Body composition will be evaluated using standardized indicators, including body mass index (BMI), waist-to-hip ratio, and body fat percentage. These assessments will be recorded at baseline, Week 16, Week 32, and at 3-month post-intervention follow-up. Bioelectrical impedance analysis (BIA) scales will be employed in these assessments, ensuring accuracy and consistency across all measurements.

Cardiovascular Fitness: Cardiovascular endurance will be measured using a 3-minute step test at both baseline, Week 16, Week 32, and the 3-month post-intervention follow-up. This test will help assess the participant's ability to recover from moderate-intensity PA, serving as an indicator of overall cardiovascular health and endurance.

Muscular Strength and Endurance: Muscular strength will be evaluated using handgrip strength tests, while endurance will be measured through a series of bodyweight exercises, including push-ups and sit-ups. These exercises target key muscle groups, providing insight into both upper body and core strength, essential for overall functional fitness.

Flexibility will be assessed using Joint Range of Motion (ROM) tests and the Sit-and-Reach Test. ROM will be measured using a goniometer to evaluate joint mobility, and the Sit-and-Reach Test will assess flexibility in the lower back and hamstrings. Participants will sit with their legs extended and reach forward, and the distance reached will serve as an indicator of flexibility, particularly in muscles critical for posture and movement efficiency.

Activity tracking: The app will also track metrics such as running speed, distance, and frequency during campus runs. These data will be recorded throughout the study, with weekly summaries providing personalized feedback to participants, helping them track their progress and stay motivated throughout the intervention.

#### 2.6.2 App usability and quality assessment

The quality and usability of the Dreamer® app will be evaluated using the Mobile Application Rating Scale (MARS) (Table 1) (25). MARS will provide a more in-depth evaluation, covering engagement, functionality, aesthetics, and information quality. Qualitative interviews will be conducted with a subset of participants to further explore their experience and satisfaction with the app.

**Table 1:** Assessment measures.

Outcomes	Instrument/ Tool	Baseline	16 Weeks	32 Weeks	3-month follow-up
Psychological and behavioral metrics					
Motivational outcomes	BREQ-3	×	×	×	×
Health-related quality of life	RAND-36	×	×	×	×
Sleep quality	Sleep condition indicator	×	×	×	×
Depression	Patient health questionnaire-9	×	×	×	×
Anxiety	Generalized anxiety disorder-7	×	×	×	×
Stress	Perceived stress scale	×	×	×	×
self-esteem	Rosenberg self-esteem scale	×	×	×	×
Social support	Oslo social support scale	×	×	×	×
Physical activity	IPAQ	×	×	×	×
Health-related physical fitness					
Muscle strength	Handgrip and push-ups/sit-ups	×	×	×	×
Body composition	Bioelectrical impedance analysis	×	×	×	×
Flexibility	ROM and Sit-and-reach test	×	×	×	×
Cardiorespiratory fitness	3-minute step test	×	×	×	×
Other outcomes					
App quality assessment	MARS		×	×	
Demographics	Questionnaire	×			

BREQ-3, behavioral regulation in exercise questionnaire – version 3; RAND-36, RAND 36-Item health survey; MARS, Mobile applications rating scale; ROM, Joint range of motion; and IPAQ, International physical activity questionnaire.

#### 2.6.3 Psychological and behavioral metrics

The mental health outcomes evaluated in this study encompass various dimensions, including motivational factors, health-related quality of life, sleep quality, and indicators of psychological well-being such as depression, anxiety, and stress. Additional measures include self-esteem and the level of social support perceived by participants (Table 1). To evaluate these aspects, this study will utilize a suite of validated assessment instruments. Intrinsic and extrinsic motivation will be measured using the Behavioral Regulation in Exercise Questionnaire (BREQ-3) (26), while health-related quality of life will be assessed with the RAND-36 Health Survey (27). Sleep quality will be evaluated through the Sleep Condition Indicator (SCI) (28), and psychological conditions such as depression and anxiety will be measured using the Patient Health Questionnaire-9 (PHQ-9) and Generalized Anxiety Disorder-7 (GAD-7) (29, 30). Stress levels will be gauged using the Perceived Stress Scale (PSS), self-esteem through the Rosenberg Self-Esteem Scale, and social support via the Oslo Social Support Scale (31–33). Participants will complete these mental health assessments at four key intervals: baseline, midpoint, the end of the intervention, and three months post-intervention. The behavior outcome of physical activity levels, measured using both self-reported and app-recorded data. Physical activity will be automatically tracked by the Dreamer® app, which records key metrics such as steps, distance traveled, and frequency of exercise. To complement the data collected through the app, participants from both the experimental and control groups will complete the International Physical Activity Questionnaire (IPAQ) at four key time points: baseline, midpoint, study completion, and three months post-intervention (34). The IPAQ will assess total PA over the previous seven days, including walking, moderate, and vigorous activities.

#### 2.6.4 Sociodemographic factors

Sociodemographic information, including age, gender, educational background, and socioeconomic status, will be collected to examine their potential moderating effects on the intervention outcomes. These factors will be measured using a pre-intervention questionnaire.

### 2.7 Data collection, security, and privacy

Data will be collected by trained assessors blinded to group allocation. Digital PA data collected through the Dreamer® application will be linked to a unique study identification number rather than participants' names in the research dataset. Direct identifiers and research data are stored separately.

Data transmitted from the application to the server will be protected using secure data transmission procedures. Stored research data will be maintained on access-controlled servers and encrypted on institutional devices. Access will be restricted to authorized research team members according to their study roles. Self-report questionnaires will be administered through a university-approved electronic survey platform.

Because participants are recruited in China, the collection, processing, storage, and use of personal information will follow applicable Chinese data-protection regulations, including the Personal Information Protection Law of the People's Republic of China, as well as institutional data-security requirements. Only de-identified or coded data will be used for statistical analysis. Identifiable information will not be included in publications or shared with unauthorized third parties.

To minimize participant burden and reduce loss from follow-up, several retention and assessment-completion strategies will be implemented. Wherever feasible, assessments will be coordinated with participants' regular PE schedules to minimize additional attendance and disruption to academic activities. Questionnaire administration and physical fitness testing may be scheduled separately when necessary to avoid excessively long assessment sessions. Appropriate rest periods will be provided during physical fitness testing. Electronic questionnaires will be used to facilitate completion, and participants will receive reminders before scheduled assessments. Where feasible, make-up assessment opportunities will be offered to participants who are unable to attend the originally scheduled session.

Research staff will provide standardized instructions and assistance during assessments to reduce avoidable missing responses. Importantly, discontinuation of the intervention will not automatically be considered a withdrawal from study follow-up. Participants who discontinue the intervention but remain willing to participate in the study will be encouraged to complete subsequent outcome assessments. Participation in, non-completion of, or withdrawal from research assessments will not affect students' PE course grades or their relationship with instructors.

### 2.8 Statistical analyses

Descriptive statistics, including means, standard deviations, and frequencies, will be utilized to summarize participants' sociodemographic characteristics, PA levels, physical health indicators at baseline. Independent t-tests will be utilized to analyze continuous variables, while chi-square tests will assess categorical variables, enabling the identification of any baseline differences between the experimental and control groups. Independent t-tests will be applied to continuous variables, and chi-square tests to categorical variables, to examine baseline differences between the experimental and control groups. In cases where significant baseline differences are identified, these variables will be controlled for in subsequent analyses.

For primary outcomes, including PA levels and health-related physical fitness metrics, repeated measures ANOVA will be used to assess changes over time (baseline, midpoint, end of intervention, and 3-month follow-up) between the experimental and control groups. The interaction effects between group (experimental vs. control) and time (pre, post, and follow-up) will be examined to determine the effectiveness of the intervention. *Post-hoc* tests with Bonferroni correction will be used for pairwise comparisons.

For secondary outcomes, such as app usability and quality outcomes, linear mixed models will be used to account for repeated measurements within individuals. The models will include fixed effects for time, group, and the interaction between group and time. Random intercepts for individuals will be used to account for individual-level variability. Effect sizes will be computed to provide a quantitative measure of the intervention's impact. For ANOVA analyses, partial eta squared (*η*p2) will be calculated to indicate the proportion of variance explained by the group, time, and their interaction. For pairwise comparisons conducted through t-tests, Cohen's d will be employed to quantify the magnitude of differences between groups or time points.

Sensitivity analyses will be undertaken to evaluate the robustness and consistency of the study findings. This will include per-protocol analyses to determine if adherence to the intervention influences outcomes. To address missing data, multiple imputation techniques will be applied, ensuring that the analysis accounts for potential biases due to incomplete observations. Sensitivity analyses will be performed to compare the results derived from imputed datasets with those obtained through complete-case analysis, ensuring the robustness of the findings.

Subgroup analyses will be conducted to explore the potential moderating influence of sociodemographic factors, including age, gender, and socioeconomic status, on the outcomes of the intervention. Interaction terms between sociodemographic factors and the group variable will be incorporated into the linear mixed models to evaluate whether the effectiveness of the app-based intervention differs across various subpopulations.

To examine the sustainability of the intervention effects at the 3-month follow-up, generalized estimating equations (GEE) will be employed. This approach accounts for the correlation of repeated measures and allows for the inclusion of participants with incomplete follow-up data. GEE will be used to assess whether the intervention group maintains improvements in mental health and behavior, and fitness metrics compared to the control group. All statistical tests will be conducted with a two-tailed approach, and a significance threshold will be established at *p* < 0.05 to determine statistical relevance. Effect sizes will be reported alongside *p*-values to provide a measure of practical significance. All statistical analyses will be conducted using IBM SPSS Statistics (Version 27.0) or R software.

### 2.9 Safety monitoring and adverse events

Participants will be screened for conditions that may contraindicate moderate-intensity exercise before enrollment. Throughout the 32-week intervention, adverse events (AEs) occurring during or in temporary association with PE classes or campus running sessions will be monitored and documented. Potential AEs include, but are not limited to, musculoskeletal pain, sprains, strains, falls, dizziness, syncope, and other exercise-related symptoms. Participants will be instructed to report such events to their PE instructor or the research team. Instructors will also actively inquire about exercise-related problems during routine follow-up.

For each AE, the date of occurrence, type of event, severity, possible relationship to intervention, management, and outcome will be recorded. Participants experiencing acute pain, injury, or other clinically concerning symptoms will discontinue the relevant activity and, where appropriate, be referred for medical assessment. Exercise participation may be temporarily suspended or modified until safe resumption is considered appropriate. Serious adverse events will be reported to the principal investigator and the institutional ethics committee in accordance with all institutional reporting requirements.

### 2.10 Ethics

Ethical approval for this study was granted by the Institutional Review Board of the Ethics Committee at Zigong Fourth People's Hospital (Approval No. EC-2025-081). All participants will provide written informed consent before enrollment.

## 3 Discussion

This RCT protocol describes the evaluation of an AI-guided, teacher-supported mHealth platform integrated within a formal university PE curriculum. Grounded in behavioral and pedagogical theory, this hybrid intervention combines digital technology with human facilitation and is designed to address persistent challenges related to student engagement and adherence in traditional PE settings. The Dreamer® application incorporates personalized feedback, real-time data monitoring, and instructor supervision—components that previous studies have suggested may support accountability and sustained motivation in technology-supported interventions (11, 12).

### 3.1 Interpretation and theoretical significance

We hypothesize that integrating the Dreamer® application with teacher-supervised PE may lead to improvements in PA, health-related fitness, and selected psychosocial outcomes compared with standard PE. This hypothesis is informed by previous evidence suggesting that mHealth interventions may support physical activity and health-related behavior change among young adults. Systematic reviews have reported short-term improvements in physical activity and related health indicators following smartphone-based interventions (35). However, engagement may decline over time when such interventions rely primarily on self-monitoring.

By embedding Dreamer® within a supervised educational framework, the present trial will evaluate whether combining digital feedback with teacher-mediated social support and pedagogical structure can support intervention adherence and longer-term behavioral maintenance. This approach is consistent with emerging perspectives on blended learning, in which digital autonomy is combined with guided instruction (36).

From a theoretical perspective, the intervention is informed by Self-Determination Theory and operationalizes several of its core constructs: autonomy through personalized app-supported feedback, competence through individualized exercise guidance and progression, and relatedness through teacher and peer interaction. These constructs have been associated with sustained motivation and PA adherence in previous research (37). The present trial will therefore provide an opportunity to examine whether integrating these theoretically relevant components within a digitally supported PE program is associated with the hypothesized behavioral and psychosocial outcomes.

### 3.2 Comparison with existing literature

Although smartphone-based interventions have demonstrated potential for promoting PA in general and clinical populations (13), relatively few have been rigorously evaluated within structured educational environments. Most previous studies have examined self-directed app use among community-dwelling adults, often without sustained behavioral reinforcement or institutional integration (12, 38). As highlighted by Wiemeyer (20), a limitation of many earlier interventions is the lack of contextual adaptation, as applications are not always aligned with the curricular goals or teaching methods of formal PE programs.

The present trial is designed to examine mHealth as an instructional adjunct rather than solely as a standalone behavior-change tool. Within the intervention, Dreamer® is integrated into the PE curriculum as a behavioral and pedagogical support tool intended to facilitate activity monitoring, self-regulation, individualized guidance, and teacher–student interaction. Previous technology-supported educational interventions have shown promising results, suggesting that educator-guided digital tools may have potential to support physical literacy and engagement (6, 36). Whether similar benefits occur within the present university PE context will be evaluated empirically in this trial.

The potential relevance of this model may also extend to blended and online sport-science education. Digital platforms that combine remote PA monitoring, individualized exercise guidance, performance feedback, and instructor oversight may provide a means of supporting selected practice-oriented components when students and instructors are not physically co-located. Previous research in a blended sport-science degree program found that supervised online workshops provided an acceptable approach to maintaining practice-oriented teaching and were associated with generally favorable learning satisfaction, although face-to-face instruction remained preferable for the acquisition of hands-on skills (39). In this context, Dreamer-like systems could potentially complement blended or distance-learning curricula by supporting remotely monitored PA and continued instructor–student interaction. However, their effectiveness for educational outcomes and practical skill acquisition in online or blended sport-science programs requires direct evaluation in future studies.

### 3.3 Practical and public health implications

If effective, the proposed intervention may provide a scalable model for integrating digital health support into university PE curricula. The widespread availability of smartphones among young adults provides an accessible infrastructure for delivering digital health interventions, while data-driven feedback systems may allow exercise guidance to be tailored to individual characteristics and activity patterns. From a public health perspective, this type of intervention is consistent with the WHO Global Action Plan on Physical Activity 2018–2030, which emphasizes the potential role of digital technologies in supporting population-level PA promotion (9).

The integration of teacher supervision within Dreamer® may also help address some of the engagement challenges reported in unsupervised digital health interventions (14). However, whether this combination of digital support and human supervision results in improved adherence or health outcomes remains to be determined. If supported by the findings, the results may inform the design of future mHealth-supported PE programs and other university-based health-promotion initiatives.

### 3.4 Strengths and limitations

A principal strength of the study design is its randomized, controlled, and longitudinal structure and its alignment with SPIRIT 2013 and CONSORT-EHEALTH reporting recommendations (22, 23). The combination of objective and self-reported measures is intended to provide a multidimensional assessment of physical, psychological, and behavioral outcomes.

However, several limitations must be acknowledged. First, this is a single-center trial conducted at one university in China. Therefore, institutional culture, PE curriculum organization, student characteristics, and local patterns of technology use may limit the generalizability of the findings to other universities, educational systems, or geographical regions. Multicenter trials involving institutions in different curricular and socio-cultural contexts may therefore be required to evaluate the transferability and scalability of the intervention. Second, because of the behavioral and technological-assisted nature of the intervention, blinding participants and instructors is not feasible (15). Awareness of group allocations may introduce performance or expectation bias. To mitigate this risk, outcome assessors and data analysts will remain blinded to allocation, standardized outcome-assessment procedures will be used, and primary analyses will follow the intention-to-treat principle. Nevertheless, residual performance bias cannot be completely excluded. Third, the repeated assessment schedule may also impose a substantial burden on participants, particularly because psychological and behavioral questionnaires are combined with physical fitness assessments at multiple time points. Although the protocol incorporates scheduling flexibility, electronic questionnaires, reminders, rest periods, and make-up assessments to support retention and completion, participant fatigue and missing follow-up data cannot be completely excluded. These factors will therefore be considered when interpreting adherence, attrition, and missing-data patterns.
